# Supplementary material for: Synthesis, Structure and Supramolecular Properties of a Novel C3 Cryptand with Pyridine Units in the Bridges
Source: Molecules. 2020 Aug 20;25(17):3789. doi: 10.3390/molecules25173789 (PMC7504419; doi:10.3390/molecules25173789)
Supplement: Supplementary file 1 [file molecules-25-03789-s001.zip › Supplementary information.docx]

***Synthesis, structure and supramolecular properties of a novel C3 cryptand with pyridine units in the bridges***

Cosmin Vasile Crişan^1^, Albert Soran ^1^, Attila Bende^2^, Niculina Daniela Hӑdade ^1^, Anamaria Terec ^1^ and Ion Grosu^1,^*

^1^ Babes-Bolyai University, Faculty of Chemistry and Chemical Engineering, Department of Chemistry and SOOMCC, Cluj-Napoca, 11 Arany Janos str., 400028, Cluj-Napoca, Romania; [kossminn@yahoo.com](http://www.chem.ubbcluj.ro/webmail/src/compose.php?send_to=kossminn%40yahoo.com); [albert_soran@yahoo.com](mailto:albert_soran@yahoo.com); [bogdan.niculina@gmail.com](mailto:bogdan.niculina@gmail.com); [asuciu@chem.ubbcluj.ro](mailto:asuciu@chem.ubbcluj.ro), igrosu@chem.ubbcluj.ro

^2^ National Institute for Research and Development of Isotopic and Molecular Technologies, 67-103 Donath str., RO-400293, Cluj-Napoca, Romania; [attila.bende@itim-cj.ro](mailto:attila.bende@itim-cj.ro)

***** Correspondence: igrosu@chem.ubbcluj.ro; +40-264-5938-33 (F.L.)

**Supplementary Information**

**Table of contents**

Atomic coordinates for the complexes **4**-G1, **4**-G2, **4**-G3 and **4**-G4 1

**Complex 4**-G1

168

O 0.17113 5.63896 5.56517

O 3.12874 8.36298 10.36356

O 7.15575 16.65010 3.25509

O 3.73523 15.34610 -0.18600

O 12.09580 5.67525 2.62361

O 9.02066 5.11011 -2.81188

N 3.03381 9.20395 1.15778

N 4.69839 9.04312 -0.49495

N 4.06840 7.12582 0.72597

N 7.16179 11.18371 6.38245

N 8.84180 10.67093 4.82119

N 8.08690 9.01719 6.33056

N 0.68265 7.00703 8.07505

N 10.74065 4.63320 -0.55459

N 6.31665 16.52411 0.50385

C 3.20274 7.90145 1.38802

C 3.82569 9.73783 0.23165

C 4.78240 7.74157 -0.21958

C 2.37870 7.30427 2.46531

C 2.51074 5.96282 2.83379

H 3.22884 5.34371 2.31106

C 1.75267 5.43562 3.85996

H 1.86492 4.40435 4.17203

C 0.84105 6.24165 4.54552

C 0.68801 7.57648 4.17893

H 0.00042 8.22748 4.69877

C 1.46049 8.09446 3.14857

H 1.36396 9.13603 2.87036

C -0.81466 6.37344 6.27047

H -1.51790 6.83821 5.57144

H -1.35316 5.62096 6.84896

C -0.25959 7.41437 7.22503

C -0.75154 8.71252 7.22599

H -1.51458 9.01067 6.51549

C -0.24028 9.61694 8.14719

H -0.60702 10.63727 8.17779

C 0.74901 9.19856 9.01950

H 1.17782 9.86528 9.75528

C 1.19092 7.88095 8.94122

C 2.25356 7.34869 9.88510

H 2.80521 6.54744 9.38989

H 1.76755 6.93197 10.77078

C 4.15017 8.72054 9.50638

C 4.11291 9.97378 8.90895

H 3.27613 10.63211 9.10298

C 5.13752 10.36404 8.06434

H 5.10254 11.32587 7.57005

C 6.21234 9.51301 7.80882

C 6.25318 8.26952 8.43635

H 7.08695 7.60889 8.24024

C 5.22912 7.87105 9.28213

H 5.25447 6.89951 9.76212

C 7.22770 9.92257 6.80597

C 7.97895 11.51261 5.38717

C 8.85838 9.43469 5.32127

C 7.86465 12.88614 4.84619

C 8.48347 13.23919 3.65303

H 9.10567 12.50844 3.15060

C 8.28477 14.49342 3.08607

H 8.74782 14.72732 2.13694

C 7.46007 15.41142 3.73263

C 6.86326 15.07777 4.94882

H 6.22618 15.80917 5.43113

C 7.05829 13.82733 5.49263

H 6.56948 13.55042 6.41738

C 7.93649 17.18249 2.20722

C 7.60168 16.67503 0.81824

C 8.63275 16.47642 -0.09577

C 8.31106 16.12490 -1.39611

C 6.97548 15.96741 -1.72687

H 6.67841 15.68091 -2.72937

C 6.01189 16.16613 -0.74205

C 4.54368 16.05067 -1.10719

H 4.45085 15.60548 -2.10379

H 4.11965 17.05693 -1.13999

C 3.78545 13.98094 -0.19134

C 4.83427 13.23877 -0.72474

H 5.68106 13.71899 -1.19310

C 4.81306 11.85430 -0.61558

H 5.63375 11.26635 -1.00846

C 3.77044 11.20272 0.03165

C 2.71615 11.96027 0.54676

H 1.90775 11.45725 1.06084

C 2.71199 13.33340 0.41997

H 1.90513 13.93258 0.82311

C 9.75202 8.45596 4.65259

C 10.48131 8.84538 3.52935

H 10.41704 9.87169 3.19700

C 11.26487 7.94238 2.83431

H 11.82203 8.27321 1.96707

C 11.31833 6.61717 3.25234

C 10.61896 6.21757 4.38769

H 10.68887 5.18530 4.71058

C 9.84345 7.13331 5.08321

H 9.29538 6.82677 5.96586

C 12.08812 5.63228 1.20319

H 11.76606 6.57366 0.76100

H 13.11882 5.45073 0.88523

C 11.20530 4.51083 0.69220

C 10.91967 3.41394 1.49707

H 11.31965 3.37103 2.50095

C 10.11589 2.41024 0.98558

H 9.86757 1.54252 1.58706

C 9.61817 2.54071 -0.30213

H 8.96569 1.78690 -0.72817

C 9.95374 3.66882 -1.03905

C 9.44381 3.80931 -2.46230

H 8.64745 3.07881 -2.64050

H 10.26142 3.58960 -3.15241

C 7.89285 5.62516 -2.23651

C 7.04142 4.93180 -1.38016

H 7.20554 3.88804 -1.14867

C 5.99337 5.60977 -0.76411

H 5.35421 5.09145 -0.05949

C 5.78082 6.96339 -0.99505

C 6.60256 7.62261 -1.91422

H 6.44053 8.67626 -2.09638

C 7.64030 6.96313 -2.53504

H 8.31649 7.48084 -3.20450

H 4.63732 15.07918 2.74290

C 4.79408 12.98805 3.19771

H 5.83037 12.95152 2.88791

H 8.17059 10.80566 1.23249

H 9.91630 10.18820 -0.41253

C 4.88711 10.53251 3.66949

C 5.86972 10.29091 2.70780

C 8.35260 9.75880 1.01377

C 9.34371 9.42196 0.09846

C 6.56656 9.08801 2.68004

C 7.60841 8.78051 1.67263

C 9.61483 8.08353 -0.16653

C 5.27266 8.32153 4.59203

C 6.25469 8.12407 3.63138

C 7.89130 7.44973 1.37375

C 8.88453 7.10034 0.48771

H 7.30268 6.65700 1.81576

H 9.05958 6.05960 0.26990

H 7.02811 6.50567 5.56724

C 4.12873 14.19983 3.12904

C 2.79882 14.27558 3.51871

C 4.16681 11.83210 3.66068

C 2.15303 13.14344 4.00347

C 2.83706 11.93908 4.07385

H 1.11115 13.21472 4.29447

C 4.59391 9.53950 4.60689

H 2.31381 11.05604 4.42520

H 2.91468 7.64815 5.79009

C 3.73105 6.99504 6.07285

H 2.46768 5.73552 7.27021

H 6.59139 4.60267 7.05056

C 6.01880 6.33568 5.91779

C 5.77856 5.26638 6.76610

C 5.00356 7.22155 5.55357

C 4.49773 5.05938 7.26308

C 3.46946 5.91913 6.90506

O 2.07886 15.43496 3.41758

O 10.58910 7.76746 -1.06840

O 4.19925 4.03381 8.12214

H 9.00192 17.02757 2.40997

H 7.73957 18.25716 2.23001

H 9.08800 15.96558 -2.13573

H 9.66574 16.59689 0.21092

H 2.62023 16.09157 2.96693

H 10.60461 6.80372 -1.19268

H 4.98875 3.50275 8.26127

H 6.05654 11.03516 1.94423

H 3.86322 9.73327 5.38322

H 6.79462 7.18938 3.63226

**Complex 4**-G2

165

O 0.06860 5.78400 5.32825

O 3.13528 8.38765 10.08894

O 8.21853 16.27132 2.60494

O 3.19831 15.61847 -0.58249

O 12.49978 5.30147 2.90343

O 9.38965 5.58284 -2.47420

N 3.06796 9.52245 1.15984

N 4.93208 9.47473 -0.27686

N 4.21693 7.49575 0.79310

N 7.27132 10.94127 6.00434

N 9.12187 10.35763 4.67706

N 8.17712 8.76576 6.13880

N 0.61562 7.08508 7.86771

N 11.08271 4.66822 -0.33366

N 5.74341 16.94181 1.39020

C 3.26210 8.22056 1.38017

C 3.93595 10.10840 0.33915

C 5.02300 8.16475 -0.03707

C 2.36925 7.57053 2.36750

C 2.45682 6.20475 2.64955

H 3.17677 5.60245 2.10968

C 1.66010 5.63419 3.62208

H 1.74223 4.58280 3.86960

C 0.76240 6.42324 4.34405

C 0.64689 7.77995 4.05462

H -0.03170 8.41737 4.60173

C 1.45181 8.34046 3.07375

H 1.39196 9.40097 2.86620

C -0.90665 6.50689 6.05874

H -1.60488 7.00237 5.37597

H -1.45511 5.74311 6.61287

C -0.34119 7.51441 7.04378

C -0.83606 8.81048 7.09170

H -1.61311 9.12551 6.40411

C -0.30375 9.69423 8.02092

H -0.66924 10.71359 8.08253

C 0.70704 9.25749 8.85840

H 1.16084 9.90895 9.59334

C 1.14088 7.93993 8.74356

C 2.21712 7.39208 9.66267

H 2.73162 6.56930 9.16294

H 1.74888 7.00518 10.57168

C 4.15299 8.67753 9.19887

C 4.15828 9.91592 8.57074

H 3.34881 10.61071 8.75600

C 5.19242 10.24605 7.71167

H 5.19342 11.19833 7.19802

C 6.23069 9.34691 7.47259

C 6.21662 8.10783 8.11230

H 7.01655 7.40524 7.91735

C 5.18513 7.77206 8.97577

H 5.17443 6.80937 9.47396

C 7.29462 9.70230 6.49884

C 8.20329 11.22562 5.09629

C 9.06007 9.13698 5.20827

C 8.19703 12.57166 4.48137

C 9.05177 12.85948 3.41180

H 9.73219 12.09199 3.06572

C 9.02404 14.09499 2.80183

H 9.67994 14.33216 1.97250

C 8.14318 15.07953 3.25765

C 7.28127 14.80513 4.31489

H 6.56306 15.53579 4.66120

C 7.31605 13.55301 4.91729

H 6.63123 13.32296 5.72349

C 7.44665 17.35370 3.07415

H 7.52078 17.44496 4.16279

H 7.90586 18.23947 2.62858

C 5.99296 17.31506 2.64843

C 4.99456 17.73116 3.51727

H 5.23652 18.01167 4.53619

C 3.68616 17.79177 3.05077

H 2.88589 18.13080 3.70038

C 3.42400 17.40431 1.74809

H 2.42129 17.42964 1.33687

C 4.49028 16.97751 0.95316

C 4.24514 16.57132 -0.48361

H 5.17942 16.22599 -0.92525

H 3.89666 17.43510 -1.05465

C 3.47125 14.29896 -0.32540

C 4.72159 13.70804 -0.48479

H 5.59008 14.28836 -0.76531

C 4.85796 12.34033 -0.28857

H 5.82283 11.86655 -0.42421

C 3.77566 11.56156 0.10364

C 2.53780 12.17383 0.29732

H 1.69932 11.57332 0.62696

C 2.38124 13.52750 0.07227

H 1.42123 14.01196 0.20658

C 10.00585 8.12987 4.66660

C 11.00800 8.53251 3.78396

H 11.11010 9.58415 3.55356

C 11.84705 7.60590 3.19334

H 12.62291 7.93139 2.51058

C 11.67674 6.25344 3.46673

C 10.69907 5.83808 4.36326

H 10.59104 4.78029 4.57261

C 9.86911 6.77555 4.96223

H 9.08949 6.46447 5.64648

C 12.57370 5.28934 1.48359

H 12.44696 6.28704 1.06060

H 13.57738 4.93881 1.22878

C 11.54663 4.35334 0.87770

C 11.13762 3.21772 1.56961

H 11.54396 3.01539 2.55168

C 10.20610 2.38334 0.97867

H 9.85910 1.49291 1.49141

C 9.71302 2.71236 -0.27599

H 8.96714 2.09433 -0.76285

C 10.17838 3.86208 -0.89884

C 9.69027 4.21644 -2.29055

H 8.83190 3.58929 -2.55331

H 10.49082 4.00969 -3.00435

C 8.25603 6.10057 -1.91417

C 7.34082 5.38445 -1.14802

H 7.46518 4.32579 -0.96586

C 6.27432 6.05522 -0.55779

H 5.57892 5.51603 0.07371

C 6.10455 7.42384 -0.72991

C 7.00752 8.11596 -1.54259

H 6.87903 9.18149 -1.67663

C 8.06703 7.46408 -2.13313

H 8.79843 7.99899 -2.72669

H 4.47252 14.63313 2.60955

C 4.76030 12.57690 3.13574

H 5.70235 12.49455 2.61181

H 8.12098 10.75053 1.49339

H 10.05973 10.25714 -0.00268

C 4.98614 10.17942 3.75841

N 6.04070 10.09858 2.93753

C 8.46573 9.73265 1.35441

C 9.54584 9.46271 0.52669

C 6.65867 8.91824 2.91495

C 7.80900 8.69971 2.01999

C 9.95950 8.14429 0.33796

C 5.25145 8.05052 4.45958

N 6.30022 7.87611 3.66538

C 8.25549 7.39260 1.84296

C 9.31315 7.11304 1.01115

H 7.74567 6.59312 2.36213

H 9.62462 6.09139 0.86398

H 6.75292 5.92096 5.01978

C 4.06976 13.77447 3.13642

C 2.84946 13.86224 3.79802

C 4.25803 11.46382 3.80479

C 2.34446 12.76549 4.49348

C 3.05144 11.57619 4.49805

H 1.39769 12.86325 5.01209

N 4.54570 9.18176 4.52758

H 2.67348 10.71157 5.03056

H 2.93771 7.65727 5.79937

C 3.63997 6.84537 5.93866

H 2.29703 5.68039 7.12850

H 6.16692 3.96295 6.45656

C 5.78639 5.85986 5.50322

C 5.46567 4.77347 6.29531

C 4.88082 6.90823 5.31889

C 4.20613 4.71403 6.88700

C 3.29044 5.74163 6.69843

O 2.10780 15.00673 3.79097

O 10.98828 7.89191 -0.51614

O 3.91074 3.62154 7.65231

H 11.03647 6.93584 -0.68285

H 2.99751 3.69158 7.94828

H 2.55228 15.65428 3.23273

**Complex 4**-G3

162

O -0.12747 5.88285 5.69389

O 3.35172 7.73536 10.50734

O 6.81920 16.46980 3.30750

O 3.63552 15.36396 -0.54684

O 12.50596 5.85452 2.82854

O 8.87900 4.78693 -2.11253

N 2.81438 9.35074 1.20356

N 4.57243 9.06411 -0.34166

N 3.87901 7.25555 1.00093

N 6.94395 10.91999 6.35057

N 8.62918 10.52931 4.75252

N 7.94401 8.79079 6.18955

N 0.69791 6.92127 8.22353

N 10.73303 4.48454 0.01741

N 6.18377 16.41414 0.44117

C 2.96896 8.06706 1.53906

C 3.64743 9.80829 0.26927

C 4.66023 7.79819 0.06452

C 2.10804 7.51709 2.60830

C 2.12314 6.14825 2.89505

H 2.75635 5.49734 2.30590

C 1.36066 5.63716 3.92141

H 1.37693 4.58179 4.16622

C 0.56828 6.48865 4.69609

C 0.54346 7.85466 4.42406

H -0.04141 8.53806 5.02455

C 1.31019 8.35499 3.37792

H 1.31210 9.41729 3.16635

C -0.99123 6.66172 6.49701

H -1.63986 7.28790 5.87556

H -1.61849 5.93233 7.01351

C -0.28567 7.50666 7.53955

C -0.70812 8.80303 7.80094

H -1.49953 9.25138 7.21039

C -0.09770 9.50633 8.83160

H -0.40931 10.51811 9.06772

C 0.91388 8.89586 9.55170

H 1.41371 9.39738 10.36968

C 1.28842 7.60144 9.20276

C 2.36688 6.86165 9.96796

H 2.80928 6.10282 9.31833

H 1.91847 6.35172 10.82397

C 4.28096 8.18521 9.59380

C 4.32690 9.54286 9.30059

H 3.63173 10.21497 9.78778

C 5.24735 10.01488 8.38005

H 5.27594 11.06491 8.11973

C 6.13779 9.14057 7.75882

C 6.10487 7.78659 8.08792

H 6.79274 7.10966 7.59818

C 5.17961 7.30628 8.99936

H 5.13962 6.25017 9.24073

C 7.06709 9.64459 6.72003

C 7.73372 11.31707 5.35351

C 8.70919 9.28226 5.21315

C 7.57727 12.69996 4.85321

C 8.27363 13.12022 3.72656

H 8.95655 12.43101 3.24623

C 8.07507 14.38645 3.19194

H 8.60345 14.66928 2.29254

C 7.15586 15.24333 3.79003

C 6.47797 14.84564 4.94511

H 5.76604 15.53121 5.38877

C 6.68274 13.58619 5.46444

H 6.13304 13.26009 6.33785

C 7.62744 17.05423 2.30656

C 7.43083 16.52377 0.89810

C 8.54751 16.27101 0.10670

C 8.35988 15.89919 -1.21427

C 7.06494 15.78880 -1.69209

H 6.87214 15.49681 -2.71839

C 6.00522 16.05090 -0.82788

C 4.58485 16.01788 -1.36382

H 4.58300 15.58253 -2.36924

H 4.22459 17.04628 -1.43472

C 3.65320 14.00465 -0.48021

C 4.66872 13.20403 -0.99500

H 5.50943 13.63106 -1.52189

C 4.62808 11.83289 -0.78092

H 5.42871 11.20174 -1.14511

C 3.59242 11.24852 -0.06254

C 2.55616 12.05976 0.40957

H 1.75025 11.60852 0.97423

C 2.57707 13.42078 0.19235

H 1.79746 14.06662 0.57852

C 9.70476 8.38057 4.58345

C 10.55419 8.85994 3.58713

H 10.47527 9.89605 3.28529

C 11.48077 8.02480 2.98741

H 12.14299 8.40133 2.21638

C 11.56239 6.69336 3.37931

C 10.72757 6.20391 4.37795

H 10.81335 5.16689 4.67872

C 9.80385 7.04695 4.97649

H 9.14602 6.67994 5.75435

C 12.34933 5.58683 1.44006

H 12.02695 6.47339 0.88967

H 13.33887 5.30443 1.07226

C 11.37666 4.45402 1.18351

C 11.21737 3.42612 2.10774

H 11.77236 3.44719 3.03633

C 10.34115 2.39811 1.80447

H 10.18411 1.58293 2.50262

C 9.66232 2.42990 0.59495

H 8.95692 1.65066 0.32759

C 9.89529 3.49033 -0.27223

C 9.22332 3.50599 -1.63215

H 8.34383 2.85229 -1.61722

H 9.92603 3.11211 -2.37010

C 7.82460 5.43453 -1.54906

C 7.11696 4.98131 -0.43835

H 7.38165 4.05950 0.06087

C 6.07051 5.74676 0.06110

H 5.52671 5.41624 0.93741

C 5.72459 6.95829 -0.52491

C 6.44112 7.39764 -1.64234

H 6.18331 8.34936 -2.08907

C 7.47312 6.64297 -2.15569

H 8.04729 6.97718 -3.01163

H 4.60300 15.10657 2.13328

C 4.91076 13.03049 2.59240

H 5.74255 12.91858 1.91137

H 7.60261 11.13725 0.40467

H 9.35104 10.81434 -1.33951

C 5.19575 10.63591 3.21427

N 6.16459 10.53575 2.30578

C 8.14087 10.20055 0.32890

C 9.11299 10.01906 -0.64368

C 6.77502 9.35275 2.23714

C 7.83637 9.17234 1.21926

C 9.77193 8.79763 -0.72256

C 5.48301 8.49628 3.87753

N 6.47791 8.30987 3.00952

C 8.51126 7.95813 1.12572

C 9.47543 7.76232 0.15191

H 8.26864 7.16241 1.81727

H 9.97194 6.80519 0.06059

H 6.75492 6.14097 4.14328

C 4.27131 14.25395 2.71338

C 3.20239 14.36916 3.58885

C 4.49741 11.93591 3.34348

C 2.77036 13.28574 4.34748

C 3.42491 12.07054 4.22479

H 1.93427 13.39371 5.02769

N 4.81461 9.63876 4.01563

H 3.11420 11.21409 4.80961

H 3.34446 8.32850 5.49224

C 3.93585 7.42335 5.50801

H 2.61719 6.38617 6.85117

H 6.07184 4.20310 5.54363

C 5.85612 6.18698 4.74523

C 5.47412 5.10623 5.52478

C 5.08798 7.35053 4.73165

C 4.30870 5.19281 6.27819

C 3.53279 6.34213 6.27549

I 2.22275 16.19737 3.76483

I 11.20691 8.49113 -2.19853

I 3.71683 3.57155 7.44336

H 8.68451 16.97698 2.58299

H 7.34870 18.11027 2.30876

H 9.20789 15.69324 -1.85824

H 9.54406 16.36468 0.52335

**Complex 4**-G4

135

O 0.32248 5.27065 6.01227

O 2.91078 8.65117 10.46204

O 7.37876 17.15466 3.30805

O 3.80081 15.21341 0.20924

O 11.82199 6.07340 2.80733

O 8.73107 4.75820 -2.45591

N 2.99157 9.06857 1.64340

N 4.54767 8.91400 -0.12368

N 3.93404 6.98114 1.07997

N 6.75673 11.61156 6.36923

N 8.52925 11.12619 4.89088

N 7.50847 9.38752 6.11297

N 0.61905 6.84058 8.42139

N 10.38827 4.56993 -0.15701

N 6.47058 16.59280 0.60235

C 3.12121 7.74887 1.80684

C 3.72601 9.60591 0.66971

C 4.63565 7.60800 0.13191

C 2.34613 7.10788 2.89002

C 2.68477 5.83031 3.34556

H 3.50978 5.30927 2.87765

C 1.99867 5.25043 4.39159

H 2.26368 4.26830 4.76496

C 0.93935 5.93139 4.99755

C 0.58034 7.19717 4.54001

H -0.23130 7.74798 4.99077

C 1.29364 7.77855 3.50023

H 1.03350 8.77147 3.15484

C -0.76677 5.89179 6.67044

H -1.52520 6.20668 5.94530

H -1.19359 5.10550 7.29531

C -0.36921 7.05813 7.55177

C -1.03421 8.27307 7.46311

H -1.83318 8.40792 6.74225

C -0.64059 9.31265 8.29544

H -1.13161 10.27824 8.24192

C 0.39366 9.09640 9.18705

H 0.74707 9.87603 9.85015

C 0.98651 7.83596 9.22066

C 2.06876 7.53321 10.23451

H 2.62663 6.64620 9.92879

H 1.61181 7.32301 11.20423

C 3.79643 8.99687 9.48069

C 4.36307 10.26670 9.59951

H 4.05791 10.89721 10.42654

C 5.28836 10.70067 8.67103

H 5.71458 11.69404 8.74542

C 5.69855 9.85840 7.63613

C 5.13092 8.59500 7.52762

H 5.43683 7.94660 6.71658

C 4.16085 8.16681 8.42421

H 3.70354 7.19711 8.28201

C 6.71114 10.31045 6.65757

C 7.65695 11.96730 5.45149

C 8.41946 9.84914 5.25467

C 7.65433 13.36552 4.97420

C 8.72106 13.86062 4.23281

H 9.58766 13.23109 4.07438

C 8.67324 15.12925 3.67640

H 9.51959 15.49133 3.10966

C 7.53279 15.91140 3.84365

C 6.46896 15.43510 4.60935

H 5.59160 16.06047 4.72290

C 6.53466 14.17617 5.17345

H 5.70154 13.78636 5.74489

C 8.11463 17.46762 2.13386

C 7.74769 16.56551 0.97503

C 8.69483 15.76600 0.34352

C 8.29135 14.96416 -0.71264

C 6.95826 14.98535 -1.09362

H 6.60753 14.36877 -1.91102

C 6.07754 15.81352 -0.40387

C 4.60671 15.88823 -0.75057

H 4.41703 15.51716 -1.76125

H 4.28729 16.92813 -0.69486

C 3.78855 13.85214 0.23289

C 4.17720 13.03712 -0.82792

H 4.51671 13.45740 -1.76362

C 4.13525 11.65834 -0.68418

H 4.46939 11.01986 -1.49268

C 3.68402 11.07393 0.49332

C 3.25683 11.90046 1.53564

H 2.91093 11.44737 2.45603

C 3.31858 13.27352 1.41358

H 3.02337 13.92577 2.22682

C 9.34410 8.87232 4.62998

C 10.13397 9.24601 3.54285

H 10.08464 10.26761 3.18681

C 10.95311 8.32411 2.91294

H 11.56039 8.62291 2.06682

C 10.99564 7.01031 3.37366

C 10.23500 6.63487 4.47518

H 10.28651 5.61203 4.82738

C 9.41160 7.56069 5.09532

H 8.79562 7.27055 5.93722

C 11.68653 5.84462 1.40967

H 11.28826 6.71887 0.89165

H 12.68876 5.64493 1.02206

C 10.79090 4.66177 1.10789

C 10.44088 3.73337 2.08132

H 10.80112 3.85038 3.09403

C 9.62805 2.67368 1.71423

H 9.32983 1.93092 2.44645

C 9.18661 2.58571 0.40268

H 8.52740 1.78533 0.08492

C 9.59410 3.55962 -0.50084

C 9.15457 3.50350 -1.95074

H 8.37514 2.74469 -2.08139

H 10.00446 3.22711 -2.57728

C 7.67969 5.37054 -1.84980

C 6.76670 4.72201 -1.02254

H 6.84770 3.66331 -0.82154

C 5.75355 5.45015 -0.41660

H 5.05890 4.95904 0.25237

C 5.62821 6.81862 -0.62921

C 6.52585 7.44949 -1.49757

H 6.43828 8.51683 -1.65665

C 7.54012 6.73704 -2.10144

H 8.26477 7.22201 -2.74424

C 6.24515 9.63727 2.85671

C 7.13510 10.02741 1.86448

C 7.91175 9.04686 1.26661

C 6.92125 7.33140 2.64113

C 7.81127 7.71900 1.65277

C 6.13450 8.30868 3.23555

H 9.19297 17.44133 2.32002

H 7.83093 18.49320 1.89635

H 9.00105 14.32417 -1.22487

H 9.72611 15.77153 0.67635

F 8.79847 9.36804 0.32034

F 8.59594 6.81935 1.06269

I 6.77884 5.35785 3.21708

F 5.25602 7.98342 4.18350

I 7.31041 12.01046 1.31700

F 5.48836 10.54270 3.48004
