# Supplementary material for: Synthesis, Structure and Supramolecular Properties of a Novel C3 Cryptand with Pyridine Units in the Bridges
Source: Molecules. 2020 Aug 20;25(17):3789. doi: 10.3390/molecules25173789 (PMC7504419; doi:10.3390/molecules25173789)
Supplement: Supplementary file 1 [file molecules-25-03789-s001.zip › 046alb_0m_a_sq-checkcif.pdf]

checkCIF (full publication check) running

checkCIF/PLATON (full publication check)

Structure factors have been supplied for datablock(s) I

THIS REPORT IS FOR GUIDANCE ONLY. IF USED AS PART OF A REVIEW PROCEDURE FOR PUBLICATION, IT SHOULD NOT REPLACE THE EXPERTISE OF AN EXPERIENCED CRYSTALLOGRAPHIC REFEREE.

```
No syntax errors found.                      CIF dictionary
Please wait while processing ....            Interpreting this report
```

Datablock: I

```
Bond precision:      C-C = 0.0016 A              Wavelength=0.71073
Cell:        a=11.4981(6)     b=15.6040(9)     c=17.0964(9)
             alpha=108.841(2) beta=105.346(2)  gamma=91.215(2)
Temperature:103 K
                   Calculated                      Reported
Volume             2780.3(3)                       2780.3(3)
Space group        P -1                            P -1
Hall group         -P 1                            -P 1
Moiety formula     C63 H45 N9 O6 [+ solvent]       C63 H45 N9 O6
Sum formula        C63 H45 N9 O6 [+ solvent]       C63 H45 N9 O6
Mr                 1024.08                         1024.08
Dx,g cm-3          1.223                           1.223
Z                  2                               2
Mu (mm-1)          0.081                           0.081
F000               1068.0                          1068.0
F000'              1068.44
h,k,lmax           15,20,22                        15,20,22
Nref               13778                           13743
Tmin,Tmax          0.988,0.990                     0.970,0.990
Tmin'              0.988
Correction method= # Reported T Limits: Tmin=0.970
Tmax=0.990 AbsCorr = MULTI-SCAN
Data completeness= 0.997         Theta(max)= 28.260
R(reflections)= 0.0351( 12753)      wR2(reflections)= 0.0845( 13743)
S = 1.053              Npar= 706
```

The following ALERTS were generated. Each ALERT has the format
test-name_ALERT_alert-type_alert-level.
Click on the hyperlinks for more details of the test.

Alert level B

```
PLAT993_ALERT_1_B No .bodd Include File with BEDE & LONE records .          ! Note
```

Alert level G

```
PLAT154_ALERT_1_G The s.u.'s on the Cell Angles are Equal ..(Note)      0.002 Degree
PLAT606_ALERT_4_G VERY LARGE Solvent Accessible VOID(S) in Structure        ! Info
PLAT869_ALERT_4_G ALERTS Related to the Use of SQUEEZE Suppressed           ! Info
```

```
   0 ALERT level A = Most likely a serious problem - resolve or explain
   1 ALERT level B = A potentially serious problem, consider carefully
```

```
    0 ALERT level C = Check. Ensure it is not caused by an omission or oversight
    3 ALERT level G = General information/check it is not something unexpected

    2 ALERT type 1 CIF construction/syntax error, inconsistent or missing data
    0 ALERT type 2 Indicator that the structure model may be wrong or deficient
    0 ALERT type 3 Indicator that the structure quality may be low
    2 ALERT type 4 Improvement, methodology, query or suggestion
    0 ALERT type 5 Informative message, check
```

---

checkCIF publication errors

Alert level A

```
PUBL003_ALERT_1_A  The contact author's name is missing,
            _publ_contact_author_name.
PUBL005_ALERT_1_A  _publ_contact_author_email, _publ_contact_author_fax and
            _publ_contact_author_phone are all missing.
            At least one of these should be present.
PUBL006_ALERT_1_A  _publ_requested_journal is missing
            e.g. 'Acta Crystallographica Section C'
PUBL009_ALERT_1_A  _publ_author_name is missing. List of author(s) name(s).
PUBL010_ALERT_1_A  _publ_author_address is missing. Author(s) address(es).
```

```
    5 ALERT level A = Data missing that is essential or data in wrong format
    0 ALERT level G = General alerts. Data that may be required is missing
```

Publication of your CIF

You should attempt to resolve as many as possible of the alerts in all categories. Often the minor alerts point to easily fixed oversights, errors and omissions in your CIF or refinement strategy, so attention to these fine details can be worthwhile. In order to resolve some of the more serious problems it may be necessary to carry out additional measurements or structure refinements. However, the nature of your study may justify the reported deviations from journal submission requirements and the more serious of these should be commented upon in the discussion or experimental section of a paper or in the "special_details" fields of the CIF. *checkCIF* was carefully designed to identify outliers and unusual parameters, but every test has its limitations and alerts that are not important in a particular case may appear. Conversely, the absence of alerts does not guarantee there are no aspects of the results needing attention. It is up to the individual to critically assess their own results and, if necessary, seek expert advice.

If level A alerts remain, which you believe to be justified deviations, and you intend to submit this CIF for publication in a journal, you should additionally insert an explanation in your CIF using the Validation Reply Form (VRF) below. This will allow your explanation to be considered as part of the review process.

Validation response form

Please find below a validation response form (VRF) that can be filled in and pasted into your CIF.

```
# start Validation Reply Form
_vrf_PUBL003_GLOBAL
;
PROBLEM: The contact author's name is missing,
RESPONSE: ...
;
_vrf_PUBL005_GLOBAL
;
PROBLEM: _publ_contact_author_email, _publ_contact_author_fax and
RESPONSE: ...
;
_vrf_PUBL006_GLOBAL
;
PROBLEM: _publ_requested_journal is missing
RESPONSE: ...
;
_vrf_PUBL009_GLOBAL
```

```
;
PROBLEM: _publ_author_name is missing. List of author(s) name(s).
RESPONSE: ...
;
_vrf_PUBL010_GLOBAL
;
PROBLEM: _publ_author_address is missing. Author(s) address(es).
RESPONSE: ...
;
# end Validation Reply Form
```

If you wish to submit your CIF for publication in Acta Crystallographica Section C or E, you should upload your CIF via the web. If you wish to submit your CIF for publication in IUCrData you should upload your CIF via the web. If your CIF is to form part of a submission to another IUCr journal, you will be asked, either during electronic submission or by the Co-editor handling your paper, to upload your CIF via our web site.

PLATON version of 22/12/2019; check.def file version of 13/12/2019

Datablock I - ellipsoid plot

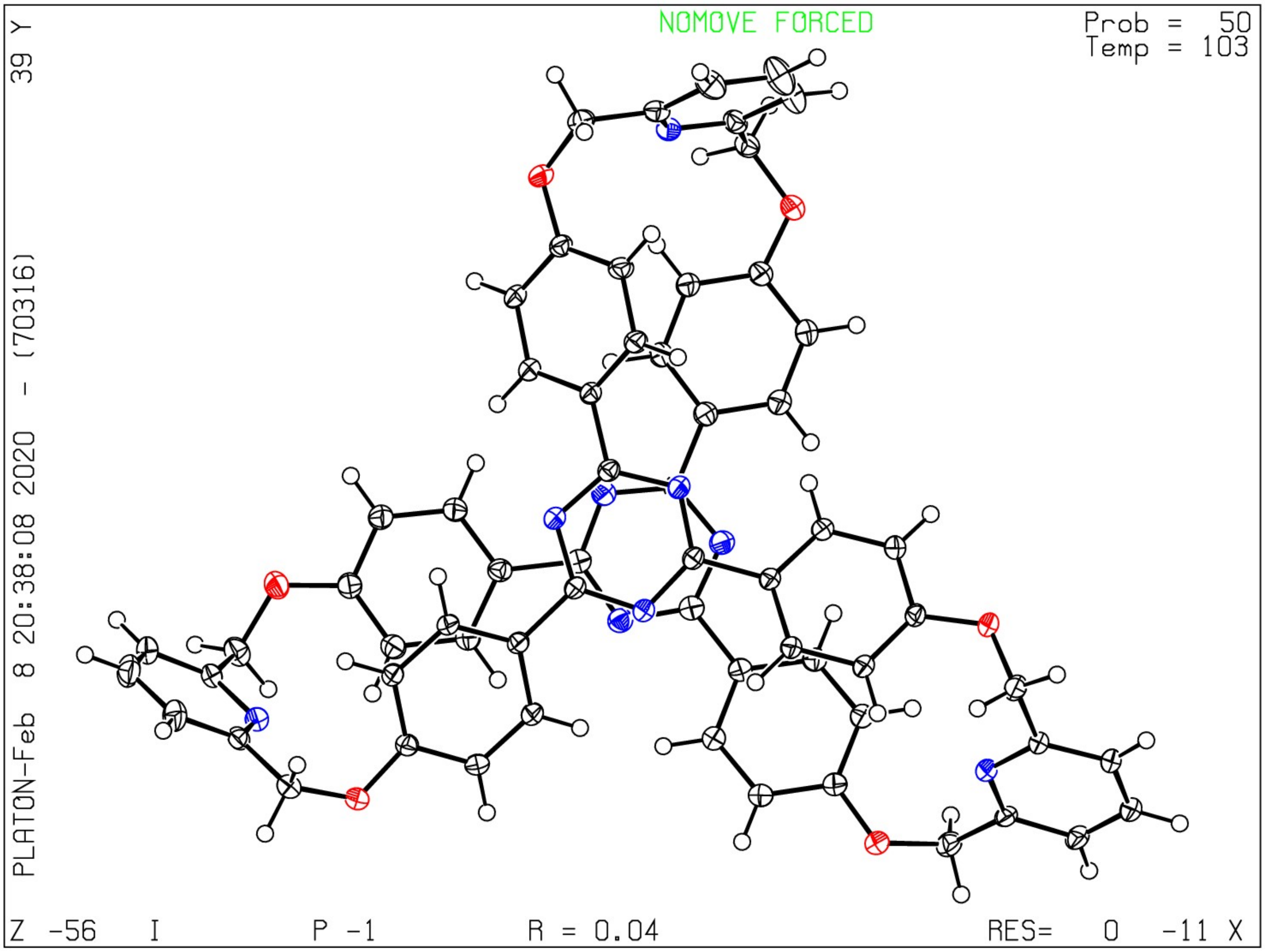

Download CIF editor (publCIF) from the IUCr
Download CIF editor (enCIFer) from the CCDC
Test a new CIF entry
